# The association of the spiritual health and psychological well-being of teachers with their organizational commitment

**DOI:** 10.1186/s40359-022-00768-x

**Published:** 2022-03-05

**Authors:** Morteza Heidari, Mohammad Ali HoseinPour, Maryam Ardebili, Sadegh Yoosefee

**Affiliations:** 1 School of Health and Religion, Qom University of Medical Sciences, Qom, Iran; 2 Islamic Azad University, Saveh Branch, Saveh, Iran; 3 Neuroscience Research Center, Qom University of Medical Sciences, Qom, Iran; 4 Spiritual Health Research Center, Qom University of Medical Sciences, Qom, Iran

**Keywords:** Spiritual health, Psychological well-being, Organizational commitment, Teacher

## Abstract

**Background:**

In line with the significance of organizational commitment, the question arises "Do spiritual health and psychological well-being optimize teachers' organizational commitment?" The purpose of this study was to determine the relationship between spiritual health, psychological well-being and the organizational commitment of high school teachers.

**Methods:**

This was a cross-sectional study in which, 346 teachers in Tehran high schools participated through multi-stage sampling. The data were collected using Ryff Psychological Well-being Questionnaire (1989), Spiritual Health Questionnaire in Iranian Society (2014) and Organizational Commitment scale of Allen and Meyer (1990), and their relationships were assessed.

**Results:**

Psychological well-being and spiritual health had positive and significant relationship with teachers' organizational commitment. Furthermore, approximately 50% of variations in organizational commitment subscales could be explained by the variables of spiritual health and psychological well-being.

**Conclusion:**

Psychological well-being and spiritual health can predict organizational commitment as the dependent variable among high school teachers.

## Background

Organizational commitment is a main issue in the field of human resource management [1]. Employee commitment may lead to important positive outcomes at individual and organizational levels [2]. The dominant model of commitment developed by Allen and Meyer, comprising three affective, normative, and continuance dimensions, but more recent models encompass two more dimensions including relevant organizational behavior and attitude to work [3]. Commitment is believed to possess both intrinsic and extrinsic dimensions. Though external motives are important and major parts of human resource management in an organization, the internal conviction is more stable and tenable, because it rooted in one’s own beliefs [4]. Therefore, it is necessary to highlight the factors affecting organizational commitment and develop it through effective educational interventions. Schools and general education system can provide the appropriate context for the growth of society in all its dimensions. Furthermore, teachers as the main actors in training and formation of different traits of human resources are the key elements in the qualitative development of education. It seems that every reform or reconstruction in the education system is condemned to failure without active contribution of teachers and their commitment which provides the resource for organizational health of the schools and the qualification of education [5]. A variety of factors have been shown to affect commitment including job satisfaction [1, 2, 6], human relations and well-being [7], burnout [8], work outcomes [9], organizational health [5], and employee’s physical health and stress reduction [10]. Organizationally committed employees are more satisfied at work, and less burned out in the organization [11, 12].

On the other hand, there are evidences denoting a relationship between spirituality and commitment along with different consequences including increased creativity, honesty, trust, personal growth and development [13]. In a more comprehensive definition, spirituality is associated with values that could be identified in the lives of everybody with regard to oneself, others, surroundings, and God [14]. It seems that integrity of person’s individual, mental, and spiritual life brings about connectedness between him and his job followed by satisfaction [15]. Spirituality has the capacity to make a balance between one's basic beliefs and organizational values and integrate them [16]. It brings a sense of unity, continuation, and understanding outstanding values in workplace [17]. Workplace spirituality promotes organizational commitment and hence organizational citizenship behavior [18]. Spirituality within the context of workplace encompasses concepts like meaningful work, interconnectedness, transcendence, and alignment of values [19]. Other evidence concerning the influence of spirituality on the attitudes and behavior of employees at the workplace, indicates that in cases of lower job satisfaction, it is compensated by spiritual factors [20].

Psychological well-being denotes a positive functioning and flourishing in life [21]. Psychological well-being is characterized by autonomy, environmental mastery, personal growth, positive relations with others, purpose in life, and self-acceptance [22]. Moreover, it is associated with consequences like positive affect, life satisfaction, sense of coherence, and optimism [23]. Well-functioning people view their lives worthwhile; they are engaged in work, having control on their work environment, feeling competent to do their work and experiencing positive relationships with others [21]. Psychological well-being entails a person’s potential for development and growth and includes feelings of personal expressiveness and accomplishment [21]. In this way, the study of the role of psychological well-being on organizational achievements seems worthwhile.

Considerable research addresses spiritual health and psychological well-being. Both the constructs are expected to be associated with commitment, especially in the affective domain [24]. Educational contexts on the other hand, are mainly influenced by teachers' commitment. Therefore, the investigation of the relationship between the variables under study in this research, can clarify some paths of promoting commitment and consequently organizational performance and accomplishment. We hypothesized that spiritual health and psychological well-being are predictive of increased organizational commitment. Therefore, this study aimed to investigate the correlation of spiritual health, psychological well-being and organizational commitment.

## Methods

This was a cross-sectional study designed to investigate the relationships of spiritual health and psychological well-being with organizational commitment. We assessed the predictive value of the multiple dimensions of the mentioned concepts.

### Sample

A total of 384 high school teachers in Tehran, Iran, received the questionnaire, and 346 returned (response rate was 90%). The sample was chosen through multistage random sampling. Out of 346 participants, 210 (61%) were female and 245 (71%) were married. Two hundred and one (58%) had a bachelor' degree, the rest (42%) possessing a master's degree. All the participants had Iranian nationality and Muslim faith.

### Data collection tools

#### Spiritual health measurement instrument in Iranian society

"Spiritual health measurement instrument in Iranian society" is a 48-item questionnaire designed and validated by Amiri et al. The instrument assesses spiritual health concentrating on one's relationships with God, oneself, and the environment in terms of insight, tendency and behavior [25]. The internal consistency for all scales and subscales were measured by Cronbach’s alpha coefficient as satisfactory with 0.70 or more.

#### Psychological well-being questionnaire

Ryff Psychological Well-being Scale was developed according to Carol Ryff's definition of wellbeing consisting of six components: self-acceptance, positive relations with others, autonomy, environmental mastery, purpose in life, and personal growth. The Short version questionnaire designed in 1989 and revised in 2002 [21, 26] contains a total of 18 questions and 6 scales. Validity and reliability of the questionnaire was shown by Van Dierendonck with the Cronbach's alpha of 0.77–0.90 [27]. The validity of its Persian translation has been reported with the internal consistency of the six subscales including self-acceptance, positive relations with others, autonomy, environmental mastery, purpose in life, and personal growth as 0.51, 0.75, 0.72, 0.76, 0.52 and 0.73 and the total Cronbach's alpha as 0.71 [28].

#### Allen & Meyer organizational commitment questionnaire

Allen & Meyer organizational commitment questionnaire is a 24 item questionnaire prepared in 1990 [29] with three dimensions of affective, normative, and continuance commitment. The reliability coefficients of this questionnaire for affective, normative and continuous dimensions were evaluated 0.85, 0.79 and 0.83 respectively [30]. The validity of the Persian version of the questionnaire was calculated and Cronbach's alpha was reported as 0.81 [31].

Data were analyzed using SPSS-20 for Pearson correlation and multiple regression analysis at a significant level of P < 0.05.

## Results

The data analysis showed the mean scores as 91.7 in organizational commitment, 89.5 in spiritual health, and 79.1 in psychological well-being. The details are shown in Table 1.

**Table 1:** The mean and standard deviation of commitment, spiritual health and psychological well-being and their dimensions (subscales)

Subscales	N	Mean	SD
Commitment			
Affective commitment	346	31.80	5.441
Continuance commitment	346	29.25	5.851
Normative commitment	346	30.69	5.786
Total organizational commitment	346	91.7	10.093
Spiritual health			
Attitude	346	91.93	9.378
Tendency	346	90.53	9.065
Behavior	346	86.20	8.046
Total spiritual health	346	89.5	5.66297
Psychological well-being			
Self-acceptance	346	13.02	1.673
Positive relations	346	13.80	1.861
Autonomy	346	13.02	1.642
Environmental mastery	346	12.71	2.302
Objective life	346	13.78	1.858
Personal growth	346	12.79	2.110
Total psychological well-being	346	79.1	4.720

Scores of organizational commitment, spiritual health and psychological well-being in relation with the demographic characteristics of the participants are presented in Table 2.

**Table 2:** Scores of organizational commitment, spiritual health and psychological well-being based on demographic characteristics of the participants

Characteristics	Commitment			Spiritual Health			Psychological Well-being		
	N	Mean	SD	N	Mean	SD	N	Mean	SD
Age									
< 30	187	91.76	–	187	89.8164	–	187	79.36	–
30–40	130	91.6	–	130	89.2103	–	130	78.68	–
40–50	27	92.37	–	27	89.8025	–	27	79.33	–
50>	2	90	–	2	84.1667	–	2	81.5	–
Total	346	91.74	–	346	89.5549	–	346	79.12	–
Sex									
Female	210	91.37	10.686	210	89.6825	5.530	210	78.95	4.884
Male	136	92.31	9.111	136	89.3578	5.877	136	79.38	4.46
Total	346	91.74	10.093	346	89.5549	5.662	346	79.12	4.72
Marital status									
Single	101	90.35	11.561	101	89.3828	5.4769	101	79.32	5.286
Married	245	92.31	9.388	245	89.6259	5.74743	245	79.03	4.475
Total	346	91.74	10.093	346	89.5549	5.66297	346	79.12	4.72
Education									
B.A*	145	91.46	9.284	145	89.2115	5.99702	145	78.94	4.416
M.A**	201	91.94	10.657	201	89.8027	5.41092	201	79.24	4.935
Total	346	91.74	10.093	346	89.5549	5.66297	346	79.12	4.72

*B.A: Bachelor of Arts; **M.A: Master of Arts

The highest commitment scores were observed in 40–50 age range. Spiritual health was highest in < 30 years, while the top scores in psychological well-being was obtained by over 50 years. Mean scores of commitment, spiritual health and psychological well-being were higher in individuals with lower degree (Bachelor). The Mean scores of psychological well-being surpassed in single participants, while commitment and spiritual well-being were higher in the married. Results of the regression analysis indicated that there was a significant effect between spiritual health, psychological well-being, and organizational commitment, (F = 169.375, p < 0.0001, R^2^ = 0.497). the results implied that approximately 50% of the subscales of the response variable (organizational commitment) are predictable by the independent variables (psychological well-being and spiritual health).

The relationship between commitment and spiritual health was calculated using Pearson correlation coefficient, while the relationship between commitment and psychological well-being dimensions was calculated through Spearman correlation coefficient. The results as presented in Table 3 indicate a positive correlation between commitments, especially continuance commitment, with spiritual health. Continuance commitment showed positive and significant correlation with the dimensions of attitude and tendency subscales of spiritual health questionnaire as well (P < 0.05). Moreover, there was positive and significant correlation between affective commitment and behavior subscales of spiritual health questionnaire (P < 0.05).

**Table 3:** Relationship between dimensions of commitment with spiritual health and psychological well-being

Scale (subscale)	Affective commitment	Continuance commitment	Normative commitment	Commitment
Spiritual health				
Correlation coefficient	0.064	0.124	0.016	0.115
Significance level	0.235	0.022*	0.765	0.032*
(Attitude)				
Correlation coefficient	0.074	0.113	0.026	0.120
Significance level	0.172	0.035*	0.631	0.025*
(Tendency)				
Correlation coefficient	− 0.051	0.109	0.028	0.052
Significance level	0.343	0.042*	0.602	0.336
(Behavior)				
Correlation coefficient	− 0.107	0.006	− 0.028	0.045
Significance level	0.047*	0.916	0.605	0.404
Psychological Well-being				
Correlation coefficient	0.519	0.313	0.380	0.680
Significance level	< 0.0001**	< 0.0001**	< 0.0001**	< 0.0001**
(Self-acceptance)				
Correlation coefficient	− 0.035	− 0.031	0.043	− 0.18
Significance level	0.571	0.571	.421	0.743
(Positive relations with others)				
Correlation coefficient	0.032	0.024	0.985	0.584
Significance level	0.584	0.651	< 0.0001**	< 0.0001**
(Autonomy)				
Correlation coefficient	0.008	− 0.120	0.047	− 0.33
Significance level	0.879	0.026*	0.384	0.535
(Environmental mastery)				
Correlation coefficient	0.989	0.015	0.18	0.538
Significance level	< 0.0001**	0.783	0.739	< 0.0001**
(Purpose in life)				
Correlation coefficient	0.033	0.984	0.27	0.583
Significance level	0.543	< 0.0001**	0.611	< 0.0001**
(Personal growth)				
Correlation coefficient	0.059	− 0.110	− 0.87	− 0.80
Significance level	0.276	0.041*	0.106	0.138
Number	346	346	346	346

**P < 0.0001; *P < 0.05

On the other hand, all dimensions of commitment showed positive and significant correlation with psychological well-being (P < 0.0001). The subscales of commitment and psychological well-being were significantly correlated between normative commitment, and positive relationship with others (P < 0.0001), between continuance commitment and autonomy (P < 0.05), between affective commitment and environmental mastery (P < 0.0001), between continuance commitment and purpose in life (P < 0.0001), and finally between continuance commitment and personal growth (P < 0.05).

Regression coefficients and constant values are presented in Table 4. As shown, psychological well-being and spiritual health have a significant relationship with commitment and can predict it as the dependent variable.

**Table 4:** Regression coefficients between spiritual health and psychological well-being with commitment

Scale	Not standardized coefficients		Standard coefficients	t	Significance level
	B	Standard error	Beta		
Constant	− 37.547	8.649		− 4.434	< 0.0001
Psychological well-being	1.490	0.082	0.697	18.157	< 0.0001
Spiritual health	0.127	0.068	0.071	1.862	0.44

## Discussion

The present study was conducted in order to illustrate the relationship that spiritual health and psychological well-being were supposed to have with the organizational commitment among high school teachers. The results showed positive and significant relationship between the above-mentioned variables as a whole and nearly half the commitment subscales were explained by spiritual health and psychological well-being. In this way, the hypothesis of the study was confirmed in general. These are in line with the findings of some previous studies. Studies have indicated positive and significant relationship between organizational commitment and spirituality in workplace and their consequent ethical behavior among the staff [32–34]. It seems that the more spirituality among teachers would cause a growth in organizational commitment and professional ethics and vice versa [34]. Another study showed that besides the relationship between the dimensions of spirituality in the workplace and organizational commitment, they are related with job satisfaction [35]. Many other studies still introduce more detailed factors affecting commitment. Employee empowerment, teamwork, and employee training are among the influencing factors [36].

To explain the significance of organizational commitment, it is best to consider the evidences emphasizing its role in the improvement of organizational performance [37, 38]. Obviously, the increased performance is the aim of many management plans and activities. Any achievement in organizations primarily, depends on the dedication of human resources and their commitments to the goals of the organization. In the field of education, goals like training creative and innovative students [39], and developing the required competencies for a good life in them cannot be achieved without commitment of the teachers to their job and the school as their organization.

Evidences indicating the relationship between spirituality and organizational commitment are noteworthy as well [32–35]. It seems that the two concepts have common components and shared values particularly in their inner nature [4]. The psychological well-being has interrelations with spiritual health [40] and thus the concepts of spiritual health, psychological well-being and organizational commitment seem interrelated and strengthening each other.

## Conclusion

Educational organizations have to care about teachers’ psychological well-being and spiritual health, so their commitment to work can make a better educational environment. The results suggest interventions or facilities for the improvement of the spiritual health and psychological well-being of the teachers. Educational leaders can consider the findings in their plans and programs because of its desirable consequences.

## Data Availability

The data supporting the findings of this study are available from the corresponding author [SY], upon reasonable request.
